# LRT: Integrative analysis of scRNA-seq and scTCR-seq data to investigate clonal differentiation heterogeneity

**DOI:** 10.1371/journal.pcbi.1011300

**Published:** 2023-07-10

**Authors:** Juan Xie, Hyeongseon Jeon, Gang Xin, Qin Ma, Dongjun Chung

**Affiliations:** 1 The Interdisciplinary Program in Biostatistics, The Ohio State University, Columbus, Ohio, United States of America; 2 Department of Biomedical Informatics, The Ohio State University, Columbus, Ohio, United States of America; 3 Pelotonia Institute for Immuno-Oncology, The James Comprehensive Cancer Center, The Ohio State University, Columbus, Ohio, United States of America; 4 Department of Microbial Infection and Immunity, The Ohio State University, Columbus, Ohio, United States of America; University of Chicago Pritzker School of Medicine, UNITED STATES

## Abstract

Single-cell RNA sequencing (scRNA-seq) data has been widely used for cell trajectory inference, with the assumption that cells with similar expression profiles share the same differentiation state. However, the inferred trajectory may not reveal clonal differentiation heterogeneity among T cell clones. Single-cell T cell receptor sequencing (scTCR-seq) data provides invaluable insights into the clonal relationship among cells, yet it lacks functional characteristics. Therefore, scRNA-seq and scTCR-seq data complement each other in improving trajectory inference, where a reliable computational tool is still missing. We developed LRT, a computational framework for the integrative analysis of scTCR-seq and scRNA-seq data to explore clonal differentiation trajectory heterogeneity. Specifically, LRT uses the transcriptomics information from scRNA-seq data to construct overall cell trajectories and then utilizes both the TCR sequence information and phenotype information to identify clonotype clusters with distinct differentiation biasedness. LRT provides a comprehensive analysis workflow, including preprocessing, cell trajectory inference, clonotype clustering, trajectory biasedness evaluation, and clonotype cluster characterization. We illustrated its utility using scRNA-seq and scTCR-seq data of CD8^+^ T cells and CD4^+^ T cells with acute lymphocytic choriomeningitis virus infection. These analyses identified several clonotype clusters with distinct skewed distribution along the differentiation path, which cannot be revealed solely based on scRNA-seq data. Clones from different clonotype clusters exhibited diverse expansion capability, V-J gene usage pattern and CDR3 motifs. The LRT framework was implemented as an R package ‘LRT’, and it is now publicly accessible at https://github.com/JuanXie19/LRT. In addition, it provides two Shiny apps ‘shinyClone’ and ‘shinyClust’ that allow users to interactively explore distributions of clonotypes, conduct repertoire analysis, implement clustering of clonotypes, trajectory biasedness evaluation, and clonotype cluster characterization.

## Author summary

Understanding the dynamic changes behind biological processes is important for determining molecular mechanisms underlying normal tissue formulation, developmental disorders and pathologies. Usually, a biological process can be characterized by identifying a trajectory, a path that goes through the various cellular states associated with the process. Since cells in different states may express different sets of genes, researchers often infer cell trajectory via capturing transcriptomics changes. Dozens of methods have been developed for cell trajectory inference, and scRNA-seq data is predominantly utilized. However, cells from different clones may exist distinct differentiation patterns, while methods based only on scRNA-seq data cannot capture this heterogeneity. T cells play a key role in the immune system, and their high antigen recognition specificity is largely determined by their TCR sequences. Thanks to the advent of scTCR-seq technology, we can now identify the group of cells coming from the same clone. This paper describes our novel computational framework, namely LRT, and demonstrates that by complementing scRNA-seq data with the clonal information from scTCR-seq data using LRT, we are able to examine the clonal differentiation heterogeneity that cannot be revealed solely based on scRNA-seq data.

## Introduction

Cell trajectory inference aims to understand how cells differentiate, which is a long-standing problem in developmental biology. Understanding how progenitor cells transform into specified functional cells can provide valuable insights into the molecular mechanisms underlying normal tissue formulation, as well as developmental disorders and pathologies [1]. Single-cell RNA-seq (scRNA-seq) data has been widely used to investigate cell trajectories. Assuming that cells in different states express different sets of marker genes, we may order cells along a differentiation trajectory via capturing differentiated transcriptional activities [2]. Based on this rationale, various computational and statistical approaches have been proposed for this purpose, namely trajectory/pseudotime analysis, where well known examples include Monocle [2] and Slingshot [3] (please see Saelens et al. [4] for a comprehensive review). While these approaches have been shown to be useful, they still suffer from intrinsic limitations. Specifically, it assumes that cells with similar expression profiles share similar developmental stages and may come from the same lineage, which might not be always true [1,5]. Besides, in the absence of clonal information, the inferred trajectory may not reflect the true clonal relationship between cells [1].

The recent advent of single-cell T cell receptor (TCR) sequencing (scTCR-seq) technology has enabled the use of TCR sequences as unique ’barcodes’ to identify clonally related cells. This is because the cells with identical paired TCR sequences usually arise from the same T cell clone due to the high dimensionality of TCR sequence space [6]. What is more, these clonal cells are often considered to be derived from the same progenitor and developmentally related [7]. Examining the abundance/proportion of clonal cells and their changes greatly benefits our understanding of adaptive immune response in health and disease [8,9]; therefore, there has been a rapid accumulation of scTCR-seq data in recent years. Accordingly, several computational and statistical approaches have also been developed, where examples include Immunarch [10] and scRepertoire [11]. However, currently available approaches mostly focus on TCR repertoire analysis, such as clonality, repertoire overlap, repertoire diversity, and gene usage analyses. The utilization of scTCR-seq data for cell trajectory analysis is still limited. In addition, although TCR data provides invaluable insights into the breadth of the antigenic response and clonal relationships between cells, it does not offer information about the functional characteristics of the T cells.

Integration of scTCR-seq data with scRNA-seq allows for the identification of T cell clones and characterize their transcriptional states at a single-cell level. This integrative analysis can reveal important insights into the clonal expansion, diversification, and differentiation of T cells in response to various stimuli, including infections, cancer, and autoimmunity. For instance, by using paired scRNA-seq and scTCR-seq data, Daniel et al. [12] discovered divergent clonal differentiation trajectories of T cell exhaustion in response to chronic viral infection, where different T cell clones follow distinct differentiation trajectories. Similarly, Khatun et al. [13] found that the during acute lymphocytic choriomeningitis virus (LCMV) infection, different naïve CD4 T cell clones showed different preference toward to either type 1 helper T cell (Th1) or T follicular helper cell (Tfh). However, despite such potential to disentangle the heterogeneity in T cell clonal differentiation trajectories, to the best of our knowledge, a computational approach to integrate scRNA-seq and scTCR-seq data for the investigation of T cell differentiation heterogeneity is missing in the literature. This is partially due to the statistical and computational challenges related to the high dimensionality of the clonotype space. Specifically, for a given human subject, 10^13^ TCR sequences are typically observed while each clonotype may only span a very limited number of cells. Hence, for effective cell trajectory inference and integration of scTCR-seq and scRNA-seq data, we need a cleverly designed computational framework that can handle these challenges.

In this paper, we propose LRT (**L**ineage inference by integrative analysis of sc**R**NA-seq and sc**T**CR-seq data), a rigorous computational framework for the integrative analysis of scTCR-seq and scRNA-seq data to study clonal differentiation heterogeneity. LRT addresses the complexity and the high dimensionality of scTCR-seq data through a clonotype clustering algorithm and effective information sharing between scTCR-seq and scRNA-seq data. In addition, to support researchers in utilizing the proposed LRT framework for their studies, we developed an R package ‘LRT’, which is now publicly accessible at https://github.com/JuanXie19/LRT. We also developed two Shiny apps, namely ‘shinyClone’ and ‘shinyClust’, and they allow researchers to interactively implement the complete LRT analysis workflow, including exploratory analysis, cell trajectory inference, clonotype clustering, differentiation trajectory biasedness evaluation and clonotype cluster characterization. These Shiny apps are provided as part of the R package ‘LRT’.

## Materials and methods

**Fig 1:**
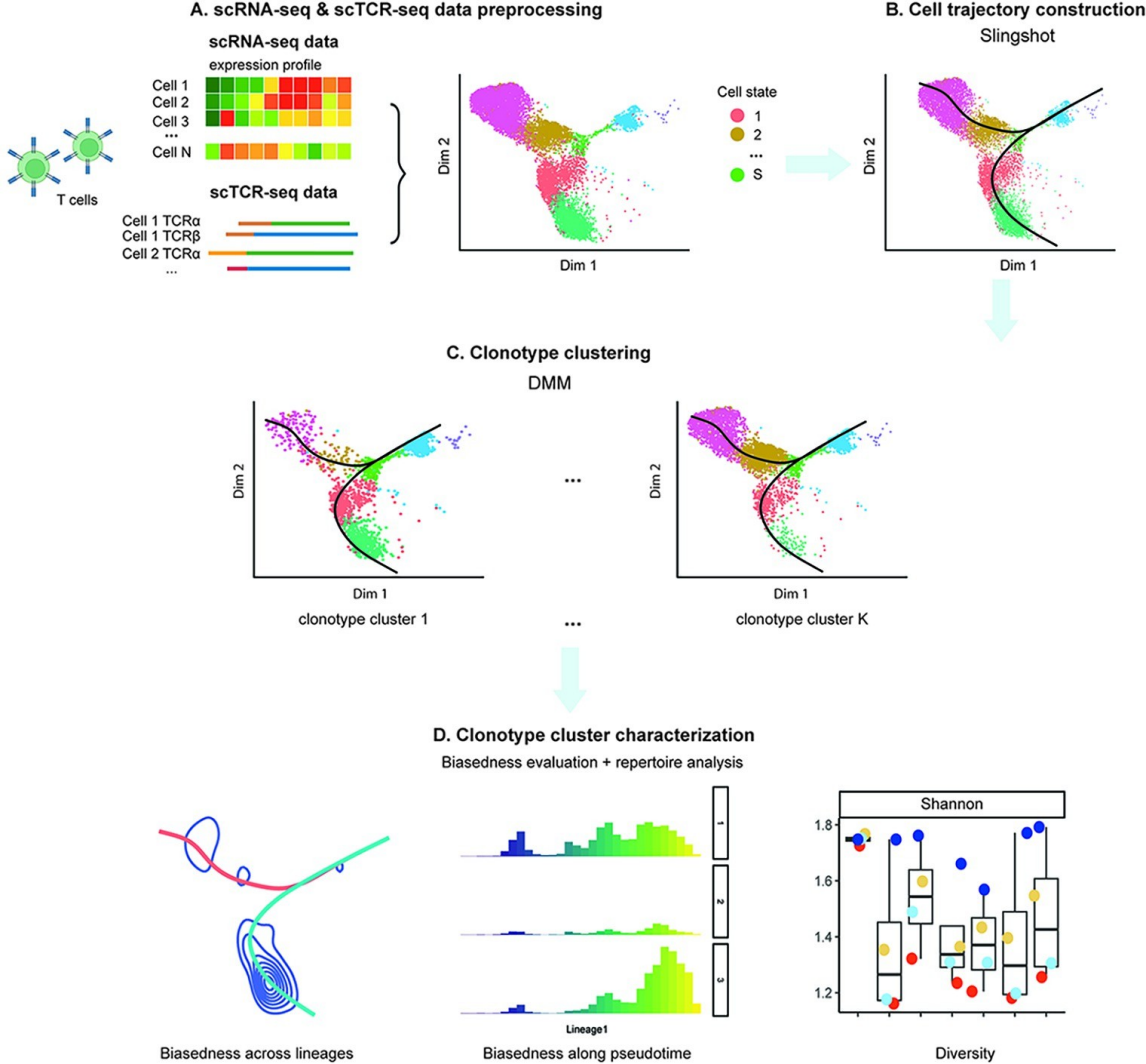
The framework of LRT. **(A)** The preprocessed scRNA-seq and scTCR-seq data are integrated based on cell barcodes matching. **(B)** Cell trajectories are first obtained using the Slingshot algorithm. **(C)** Clonotypes with similar cell cluster composition are grouped using the Dirichlet multinomial mixtures (DMM) model. **(D)** For each clonotype cluster, the distributional bias of clones along the trajectories is evaluated via permutation tests. The repertoire is characterized in terms of clonal expansion and diversity, the top-ranked clonotypes, and V and J gene usage patterns, among others.

The workflow of the ‘LRT’ framework is shown in **Fig 1**, which starts from the integration of scTCR-seq and scRNA-seq data (**Fig 1A**). Essentially, scTCR-seq and scRNA-seq data are paired via barcode matching, and TCR sequence information is added as metadata of the scRNA-seq data. Using this integrated dataset, LRT applies the Slingshot algorithm [3] to infer the overall cell trajectory (**Fig 1B**). Next, LRT identifies clusters of clonotypes using a Dirichlet multinomial mixture model (DMM) (**Fig 1C**). The biasedness along the overall trajectory for clones in each clonotype cluster is evaluated by using permutation tests. Besides, the identified clonotype clusters are characterized via repertoire analysis, top-ranked clonotypes identification, and V-J gene usage pattern analysis (**Fig 1D**).

### Cell trajectory estimation

To characterize the potential process of T cell functional changes, LRT applies the Slingshot algorithm [3] on the scRNA-seq data (see the Section ‘Software implementation’ for more details). Specifically, a weighted graph is constructed, with nodes being cell-cluster-specific centers and edge weights represent the Euclidean distances between the nodes on the low-dimensional space, such as Principal Component Analysis (PCA) or Uniform Manifold Approximation and Projection (UMAP) space [14]. Note that both cell clusters and low-dimensional space in this step are defined based on scRNA-seq data. A cluster-based MST [15] is then constructed, and a simultaneous principal curves method as described in Slingshot is applied to fit smooth branching curves [3]. The obtained smoothing curves are interpreted as trajectories and the arc length between the starting point of the curve and the projection of cell on the curves is treated as pseudotime.

### Clonotype clustering

While a clonotype of our ultimate interest, scTCR-seq data often suffers from high dimensionality and sparsity issues, i.e., we often have a large number of clonotypes, each of which spans only a few cells. Such high dimensionality and sparsity remain challenges to reliably investigate the distributon of clones along the trajectories and their heterogeneity. In order to address these challenges, LRT conducts a clustering analysis to group similar clonotypes, where similarity is defined based on cell distribution across cell clusters/states. The rationale behind is that cells from different clones may have different preference towards a particular cell state, and thus exhibit diverse distribution across cell states. In LRT, the clonotype clustering is implemented via a Dirichlet multinomial mixtures (DMM) method proposed by [16], which can deal with the sparse and heterogenous nature of the frequency matrix for the clonotypes. In addition, because the DMM method is based on a Bayesian framework, it provides uncertainty quantification and posterior probabilities for cluster memberships of clonotypes, which allow us to prioritize clonotypes for downstream analyses and experimental validations.

Let’s denote *X* as a matrix of frequencies, in wich elements *x*_*ij*_ being the obseved frequency of cells that belong to cell state *j* for clonotype *i*, with *i*∈[1:*C*], *j*∈[1:*S*]. We further denote the rows of the matrix that give the cell distributions across cell states for each clonotype by *x*_*i*_. We assume that each clonotype is generated from a multinomial distribution with parameter vector *p*_*i*_. The elements of *p*_*i*_, *p*_*ij*_, are the probabilities that a cell from clonotype *i* belong to the cell state *j*. Under this setting, the likelihood of observing the cell distribution for a clonotype *i* is given as follows:

$$L_{i}(x_{i}|p_{i})=J_{i}!\prod_{j=1}^{S}\frac{p_{ij}^{x_{ij}}}{x_{ij}!}$$

where $J_{i}=\sum_{j=1}^{S}x_{ij}$ are the total number of cells in clonotype *i*. The total likelihood is the product of the clonotype likelihoods:

$$L(X|p_{1},\ldots ,p_{C})=\prod_{i=1}^{C}L_{i}(x_{i}|p_{i})$$

As regard to the prior distribution for the multinomial parameter vectors *p*_*i*_, a natural choice would be the Dirichlet distribution, which is conjugate to the multinomial. Yet considering that different clonotypes might have significantly different cell distributions, a mixture of *K* Dirichlets is adopted instead of assuming a single Dirichlet prior to deal with cell state composition heterogeneity. Specifically, for each clonotype *i*, we assume that it belongs to one of *K* clusters. Hence, the prior is:

$$P(p_{i}|Q)=\sum_{k=1}^{K}Dir(p_{i}|\alpha_{k})\pi_{k}$$

where *Dir*(*p*_*i*_|*α*_*k*_) represent the Dirichlet distribution with parameter *α*_*k*_, *π*_*k*_ is the mixture weight, and *Q* = (*α*_1_,…,*α*_*K*_,*π*_1_,…,*π*_*K*_) is the hyperparameters. We further assume that *α*_*ik*_~*Γ*(*η*,*ν*). We use a *K*-dimentional binary latent indicator vector *z*_*i*_ to represent the membership of *i*-th clonotype. For the parameter interfence, a EM algorithm is employed, and the number of Dirichlet compoments, *K*, is determined through the Laplace approximation to the posterior probability. For more details about parameter estimation and model selection, please refer to [16]. After determining the number of mixture component, i.e, the number of clusters *K*, we infer which cluster an individual clonotype belong to by finding the value of *k* that maximizes the posterior probability of membership:

$$k=arg\underset{k^{\prime }\in \{1,\ldots ,K\}}{max}P(z_{ik^{\prime }}=1\vee x_{i},\hat{Q})$$

### Statistical inference for the trajectory bias

In the integrative analysis of scRNA-seq and scTCR-seq, we are often interested in the distributional biasedness along the trajectory for clones. The DMM method described in the previous section allows us to obtain *K* clonotype clusters, each with distinct composition (i.e., cell distribution across cell states), which likely reflects such distributional bias. However, researchers can be benefited with a rigorous hypothesis testing that quantifies statistical significance of the distributional bias. For this purpose, we developed 3 different permutation tests to evaluate the distributional bias along the trajectory for clones. We note that we might want to evaluate such distributional bias at the clonotype level. However, as also discussed in the previous section, scTCR-seq data often suffers from high dimensionality and sparsity issues and these can make such permutation tests less stable and reliable. Hence, based on the similar rationale as in the previous section, we conducted these permutation tests at the clonotype cluster level.

Hypothesis testing procedures have three components: definition of the null hypothesis (*H*_0_), definition of the test statistics, and derivation of the null distribution to determine rejection of *H*_0_. First, here we consider three different types of distributional biases in the sense of (i) the biased use of different trajectories; (ii) the specific localization in a certain differentiation stage vs. the more dynamic changes across the differentiation stages; and (iii) the preference for earlier vs. later differentiation stages. We note that cells are often not uniformly distributed between different trajectories or along differentiation stages. Given this, instead of considering the uniform distribution as *H*_0_, we use the global patterns as the null hypothesis and evaluate whether a specific clonotype cluster shows significantly different patterns compared to this global distribution.

Second, in the sense of test statistics, for the case of the hypothesis testing (i), we would like to evaluate whether clones were preferentially distributed towards a particular lineage or not. For this purpose, we use the test statistic $R_{klm}={log}_{2}\left(\frac{cells\in lineagel}{cells\in lineagem}\right)$, which is the log2-transformed ratio of the number of cells in lineage *l* to the number of cells in lineage *m* for clonotype cluster *k*. Note that *R*_*klm*_ has nice interpretation: if *R*_*klm*_ is significantly larger than 0, then it indicates the preference for lineage *l*, while if *R*_*klm*_ is significantly smaller than 0, then it indicates the preference for lineage *m*. For the case of the hypothesis testing (ii), the specific localization in a certain differentiation stage vs. the more dynamic changes across the differentiation stages can be formulated as the degree how evenly cells are spaced along the trajectory path. We use the interquartile range (*IQR*) of the pseudotime of a clonotype cluster as the test statistic for this purpose based on the rationale that *IQR* will become smaller as we have more specific localization in a certain differentiation stage. This test is also applicable to the case when there are multiple lineages. For the case of the hypothesis testing (iii), if the cells are not evenly spaced along the trajectory path (i.e., if the hypothesis testing (ii) is rejected), then we want to check whether earlier stage along the trajectory or the latter stage is preferred. To answer this question, we use the median pseudotime as the test statistic because it can represent where the majority of cells belonging to a specific clonotype cluster are located over the pseudotime. Specifically, if the observed median pseudotime is significantly smaller/larger than the global median, we may say that cells prefer earlier/later stage.

Finally, to derive the null distribution, we use permutation test approaches. Specifically, in each permutation set, we randomly shuffle the clonotype cluster membership for the cells while maintaining the size of each clonotype cluster. Then, for each permutation set, we calculate the test statistics described above and obtain the distribution of these null test statistics. Finally, in the case of the hypothesis testing (ii), we obtain the *p*-value as the proportion of permutation sets of which test statistics is smaller than the test statistic observed in our dataset (i.e., 1-sided hypothesis testing) because we are interested in the smaller IQR, which indicates the specific localization in a certain differentiation stage. On the other hand, in the case of the hypothesis testing (i) and (iii), we obtain the *p*-value as the proportion of permutation sets of which test statistics is either smaller or larger than the test statistic observed in our dataset (i.e., 2-sided hypothesis testing) because both directions are of interest. For example, the smaller and larger test statistics for the hypothesis testing (i) mean preference for lineage *m* and lineage *l*, respectively. Similarly, the smaller and larger test statistics for the hypothesis testing (iii) mean the preference for earlier vs. later differentiation stages, respectively.

### Clonotype cluster characterization

To characterize the clonotype clusters, first, LRT also quantifies the magnitude of clonal expansion, migration potential and state transition potentials of clonotypes by utilizing the STARTRAC framework [17]. Both clonotype level and clonotype cluster level indices are calculated. For the details, please refer to **Section A in S1 Text**. Second, a range of repertoire analysis is conducted for each clonotype cluster. Specifically, LRT calculates several diversity indices including the Shannon index [18], which characterizes richness and evenness of the TCRs in a clonotype cluster. To understand the clonal expansion behaviors of the clones, LRT also analyzes the relative clonal space occupied by the clonotypes of a given frequency range as well as top-expanded clonotypes. Third, given that biased usage of specific genes may indicate the alterations in the repertoire, LRT calculates the V and J gene usages of the TCR *β* chain [11]. Finally, we do *de novo* motif analysis to identify the motifs for the amino acid sequences of the TCR *β* chain using the GLAM2 tool within the MEME Suite [19], based on the top-ranked clones with the default setting. Here clonotypes are ranked in each clonotype cluster based on their membership assignment posterior probability, $P(z_{ik}=1\vee x_{i},\hat{Q})$, and clone size. Specifically, a clonotype is likely to be the higher ranking one if it has higher posterior probability and larger clone size.

### Software implementation

The aforementioned LRT framework and the whole analysis workflow were implemented as an R package ‘LRT’ and it is now publicly accessible at https://github.com/JuanXie19/LRT. The LRT software takes both scRNA-seq and scTCR-seq data as input. It accepts scRNA-seq data in the form of a Seurat object [20]. Such integration with Seurat not only allows LRT to be easily integrated into an existing scRNA-seq analysis workflow, but also utilizes Seurat’s powerful features, e.g., correcting batch effects and variations due to technical factors (e.g., sequencing depth) [20,21]. The Seurat object needs to contain cell cluster labels and low-dimensional embeddings for each cell, such as PCA or UMAP. In the case of scTCR-seq data, it requires a data frame with at least two columns: one column with cell identifiers and another column with TCR sequence information, for which users can provide either amino acid or nucleotide sequences of TCR α, β, or paired α-β chain CDR3 region.

### Data preprocessing

LRT integrates scTCR-seq data with scRNA-seq data by attaching the TCR sequence information to the metadata of the Seurat object. LRT also provides some basic quality control functionalities, e.g., checking whether the cell identifiers from the Seurat object match those in the TCR data frame. After the integration, users will get an S4 object containing the updated Seurat object, which is the input for clonotype exploratory analysis and trajectory inference.

### Shiny apps for interactive visualization and analysis: shinyClone and shinyClust

We developed two Shiny apps, *shinyClone*, and *shinyClust*, for interactive and dynamic data exploration and integrative analysis of scRNA-seq and scTCR-seq data.

### shinyClone

**Fig 2:**
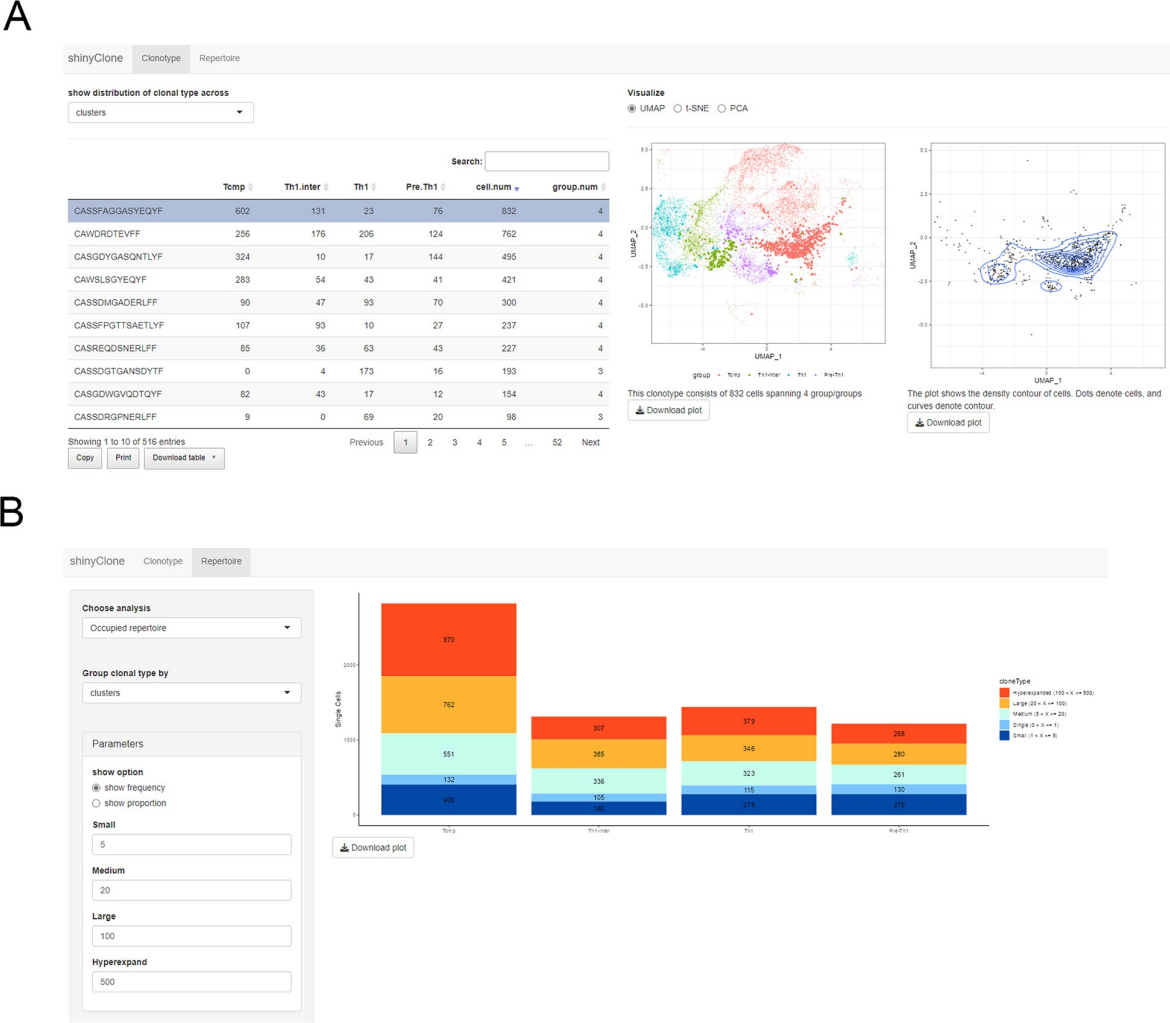
shinyClone, a Shiny app for exploratory analysis of clonotypes. **(A)** The ‘Clonotype’ tab allows users to implement various exploratory analyses of clonotype distributions. **(B)** The ‘Repertoire’ tab provides various TCR repertoire analysis functionalities.

*shinyClone* is a Shiny app for exploratory analysis of clonotypes (**Fig 2**). It provides commonly used repertoire analysis functionalities and lets users intuitively explore how each clonotype is distributed on the reduced dimensional space generated based on scRNA-seq data. *shinyClone* consists of two tabs, one for exploratory analysis of clonotype distribution (tab ‘Clonotype’) (**Fig 2A**) and the other for repertoire analysis (tab ‘Repertoire’) (**Fig 2B**). First, on the ‘Clonotype’ tab, users can find a table of clonotypes, along with the relevant information, including (i) how many cells are associated with each clonotype; and (ii) how these cells are distributed among groups (e.g., cell clusters, samples, conditions, or any other groups as available in the metadata of Seurat object) (**Fig 2A**, left). Users can sort the table by clicking each column name, e.g., to check the most/least abundant clonotypes. By clicking each clonotype in this table, the user can check the distribution of cells associated with each clonotype on the reduced dimensional space, for which the user can choose among UMAP, t-SNE, or PCA for the dimension reduction (**Fig 2A**, middle). A cell density plot is also displayed for clones spanning more than 10 cells (**Fig 2A**, right). Both plots can be downloaded in the PDF file format by clicking the “Download” button. Second, the ‘Repertoire’ tab provides several common repertoire analysis functions using the R package ‘scRepertoire’ [11], where examples include clonal diversity, clonal homeostasis, clonal proportion, and clonal overlap analyses (**Fig 2B**).

### shinyClust

**Fig 3:**
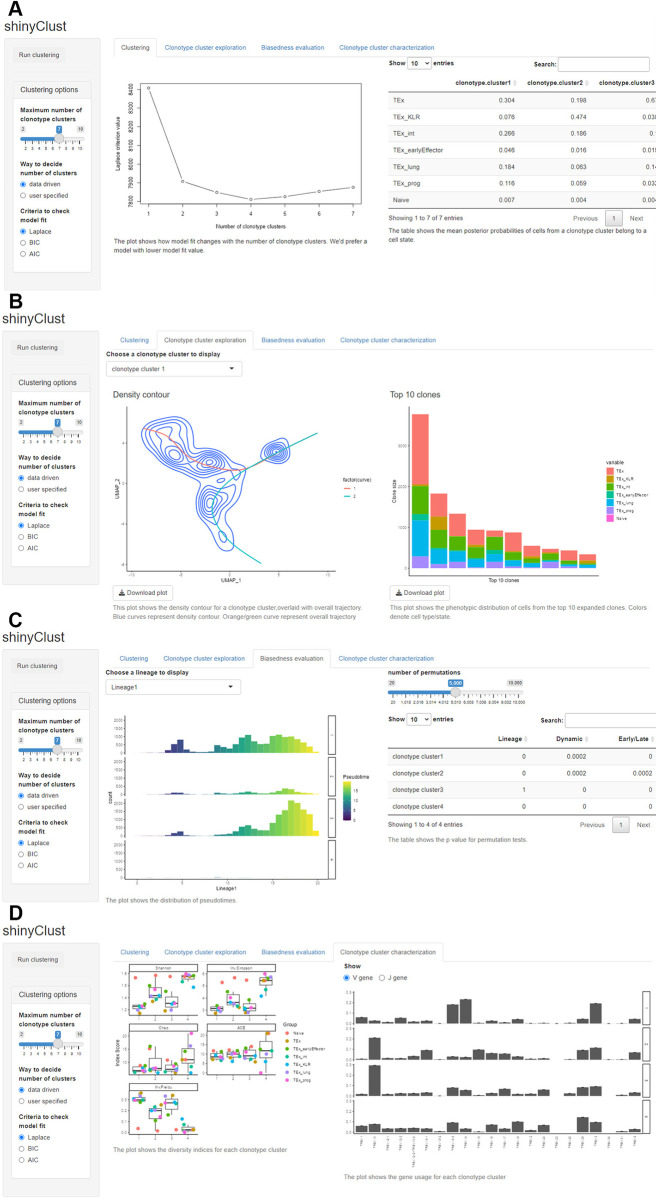
*shinyClust*, a Shiny app for clonotype clustering and marker identification. **(A)** The ‘Clustering’ tab shows the summary of clonotype clustering. **(B)** The ‘clonotype cluster exploration’ tab displays the density contour of cells and distribution of top-expanded clones in the selected clonotype cluster. **(C)** The ‘Biasedness evaluation’ tab shows the results of permutation tests to evaluate trajectory biasedness. **(D)** The ‘Clonotype cluster characterization’ tab shows the characteristics of the selected clonotype cluster.

*shinyClust* is another Shiny app that allows users to interactively and dynamically implement the clonotype clustering analysis and clonotype cluster characterization (**Fig 3**). *shinyClust* consists of four tabs, ‘Clustering’ (**Fig 3A**), ‘Clonotype cluster exploration’ (**Fig 3B**), ‘Biasedness evaluation’ (**Fig 3C**) and ‘Clonotype cluster characterization’ (**Fig 3D**). First, the ‘Clustering’ tab allows users to interactively implement clonotype clustering (**Fig 3A**). Users can specify the range for the number of clusters and choose either Laplace, AIC or BIC as a model fit criterion. This page shows a plot of the chosen metric for different numbers of clusters to assist users to determine the optimal number of clusters. The Bioconductor R package ‘DirichletMultinomial’ [22] is used for the DMM implementation. Second, on the ‘Clonotype cluster exploration’ tab, upon choosing a particular cluster to display, users can check the distribution of the cells from a specified clonotype cluster along the overall trajectory (**Fig 3B**, the plot on the left), as well as the phenotypic distribution of cells for the top-10 expanded clones in the cluster (**Fig 3B**, the plot on the right). Next, on the ‘Bias Evaluation’ tab shows the histogram of pseudotime for each clonotype clusters, along with the permutation test *p*-values evaluating trajectory biasedness (**Fig 3C**). Finally, on the ‘Clonotype cluster characterization’ tab, *shinyClust* updates (i) diversity plot, which shows various diversity indices (**Fig 3D**, the plot on the left); and (ii) a barplot, which shows the V and J gene usage patterns for the TCR *β* chain of the clones (**Fig 3D**, the plot on the right).

## Results

### LRT identified groups of clonotypes with distinct differentiation trajectory bias in CD8^+^ T cells

**Fig 4:**
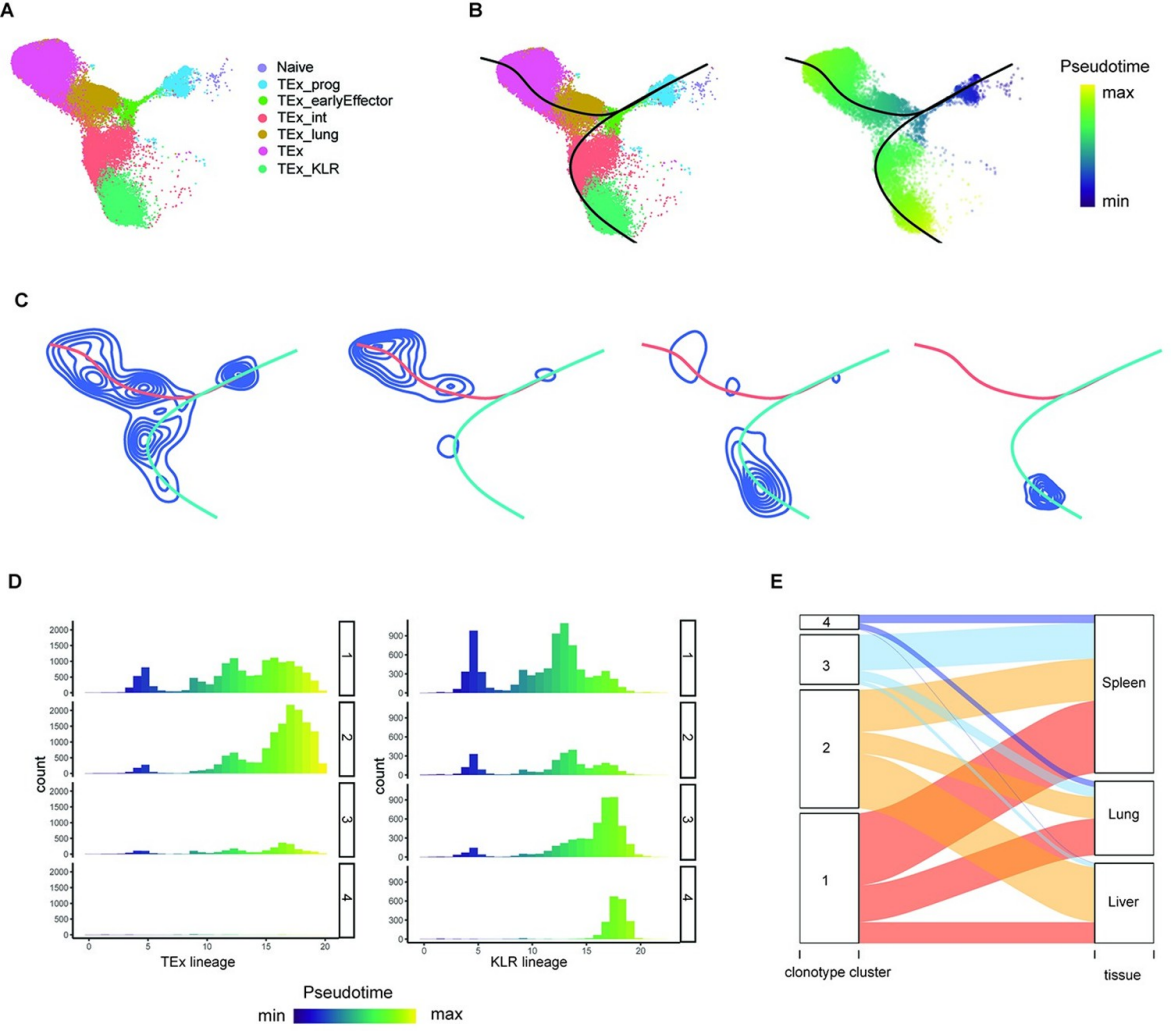
LRT analysis of the CD8^+^ T cells from the mouse chronic LCMV infection data at D21. **(A)** UMAP of the cells. Colors represent T cell subsets. **(B)** Trajectory analysis. Left: divergent trajectories inferred by Slingshot; Right: pseudotime of cells. **(C)** Density contour plots for the identified clonotype clusters overlayed with overall trajectories. Blue curves denote the cell density contour, and colored curves denote overall trajectories. **(D)** Histogram of pseudotime for cells in each clonotype cluster. **(E)** Alluvial plot showing the flows of cells in each clonotype cluster across cell state and across tissues.

To demonstrate the utility of LRT, we analyzed the scTCR-seq and scRNA-seq data generated from the antigen-specific CD8^+^ T cells during chronic lymphocytic choriomeningitis virus infection [12] (GEO accession number: GSE188670). We used Seurat (v4.2.0) to load the data and create a Seurat object for the scRNA-seq data and used the scTCR-seq data from the provided raw data (GSE188666_RAW.tar). The authors already preprocessed the scRNA-seq data and also provided cell annotation and UMAP coordinates in the metadata (GSE188666_SCrna_LCMV_metadata.tsv.gz). Hence, we did not implement additional preprocessing. The original data contains cells from both acute and chronic infections collected on Day 8 and 21, which totals more than 70,000 cells. For the illustration purpose, here we focused on a subset of the data from chronic infections collected at D21. In the case of the scTCR-seq data, only chains that were annotated as full-length and functional were retained. If a cell contained multiple full-length functional sequences for a given chain, the sequence supported by the largest number of unique molecular identifiers (UMIs) was retained for analysis. After merging scRNA-seq and scTCR-seq data, we further filtered clonotypes (defined by paired TCR *α* and *β* chain sequences) with less than 5 cells to reduce the impact of noise for downstream analysis. After filtering, we have a total of 893 clonotypes with 44,041 cells. According to the provided annotation, these cells are subsets of exhausted T cells (Tex), including naïve, progenitor Tex (TEx_prog), early effector exhausted Tex (TEx_eeff), killer cell lectin-like receptor-expressing cytotoxic Tex (TEx_KLR), lung terminal exhausted cells (TEx_lung), and terminal exhausted Tex (TEx) (**Fig 4A**)

To investigate the cell differentiation paths, we applied the Slingshot algorithm [3] to perform trajectory analysis. Consistent with [12], divergent differentiation trajectories were identified (**Fig 4B**). Specifically, the trajectory path went through naïve, progenitor, early effector states, and then at the intermediate state, cells bifurcate into either terminal exhausted state or KLR state. Next, to investigate the heterogeneity among clones in terms of clonal differentiation biasness, we grouped clonotypes based on their phenotypic distribution patterns using the DMM method and identified four clonotype clusters based on the Laplace criteria (**Fig A and Table A in S1 Text**). Generally, the posterior probability for a cell in a clone to adopt TEx phenotype is much higher than the probability of adopting TEx_KLR phenotype for clones in clonotype clusters 1 and 2 (0.304 vs 0.076, and 0.670 vs 0.038, respectively), while the case is the opposite for clonotype clusters 3 and 4. The distribution of these clusters along the overall trajectory exhibited distinct phenotypic patterns along the differentiation path (**Figs 4C and B and C in S1 Text**). Clonotype cluster 1 consists of divergent clones, with cells that acquired both TEx and TEx_KLR phenotypes although the density along the TEx lineage still seems to be higher than that along the TEx_KLR lineage. Besides, clonotypes in this clonotype cluster also have abundant early-stage naïve cells, which are largely missing in other clonotype clusters. Clonotype clusters 2, 3 and 4 show strikingly higher proportions of later-stage cells. Moreover, clonotype clusters 3 and 4 are dominated with TEx_KLR while cluster 2 mostly adopted TEx phenotype, i.e., they are most likely to be TEx_KLR-biased and TEx-biased, respectively. Compared with clonotype cluster 3 in which other phenotypes also exist, cells in clonotype cluster 4 almost exclusively adopted TEx_KLR phenotype. Permutation tests was further conducted to evaluate the biasedness of clones towards either TEx lineage or TEx_KLR lineage, which indicates that clones in clonotype clusters 1 and 2 are TEx biased, while clones from clonotype clusters 3 and 4 are TEx_KLR biased (hypothesis testing (i) *p*-values = 0 for all these 4 clonotype clusters). Besides, clonotype cluster 2 is more biased compared to clonotype cluster 1, and clonotype cluster 4 is more skewed in relative to clonotype cluster 3 (**Fig D in S1 Text**).

The distribution for cells in each clonotype cluster over the pseudotime provides additional insights regarding the bias of clones towards differentiation stage for clonotype clusters (**Fig 4D**). Along the TEx lineage, clonotype cluster 2 was significantly localized in a specific differentiation stage (hypothesis testing (ii) *p*-value = 0; **Fig E** the first row **in S1 Text**). Specifically, cells from clonotype cluster 2 peaked late along the TEx lineage (hypothesis testing (iii) *p*-value: 0; **Fig F** the first row **in S1 Text**). In contrast, along the TEx_KLR lineage, most clonotype clusters except cluster 2 were significantly localized (hypothesis testing (ii) *p*-values for clonotype clusters 1–4: 0, 0.217, 0, 0; **Fig E** the second row **in S1 Text**). Specifically, cells from clonotype cluster 1 peaked at earlier pseudotime while cells from clonotype clusters 3 and 4 peaked at later pseudotime along the KLR lineage (hypothesis testing (iii) *p*-values for clonotype clusters 1, 3, 4: 0; **Fig F** the second row **in S1 Text**).

Divergent and biased clones also presented different state transition potentials. Specifically, we found that KLR-biased clones usually do not appear in liver: only a very small proportion of cells from clonotype cluster 3 show in liver, and the proportion of liver cells in clonotype cluster 4 is even negligible (**Fig 4E**). Our findings are consistent with [12], which reported that TEx_KLR clones are depleted in the liver microenvironment. The clonotype cluster level and pairwise tissue migration scores also supported this point: the STATRAC migration score for clones in clonotype clusters 3 and 4 are just a half of the values for clusters 1 and 2, and clones in cluster 4 have close to zero mobility between liver and other tissues (**Fig G in S1 Text**). Consistent with the fact that majority of cells in cluster 4 are TEx_KLR cells, we observed very low pairwise state transition scores for clones in clonotype cluster 4. Clones in cluster 1 generally have medium-to-high transition potentials across most states. This is not surprising, given that a fair proportion of cells falls in each cell state. The transition between TEx_int—TEx_lung and TEx_int—Tex_KLR are noticeably high for clonotype clusters 2 and 3, respectively, and again as expected considering that they are TEx-biased and TEx_KLR-biased.

**Fig 5:**
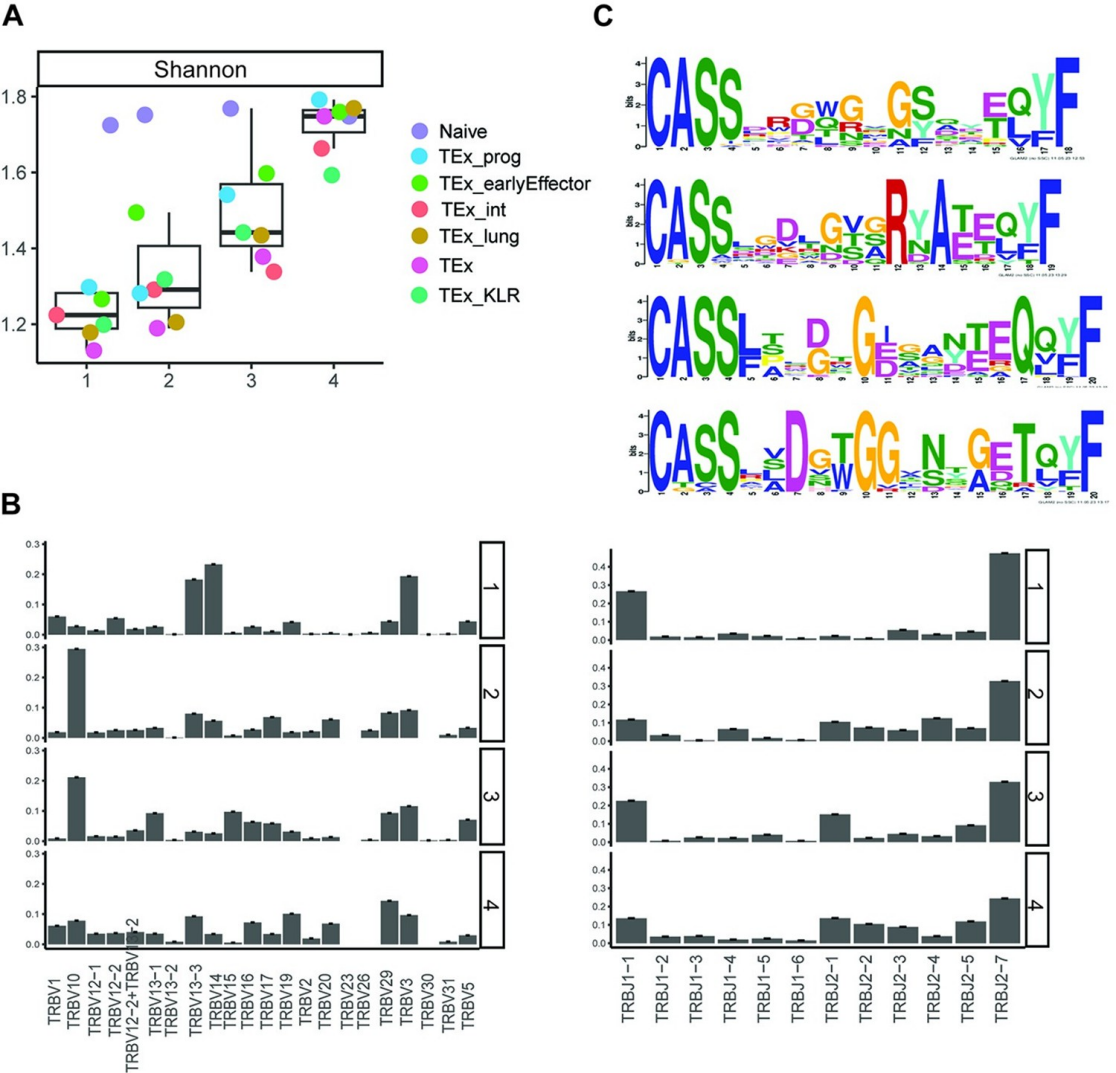
LRT analysis of the CD8^+^ T cells from the mouse chronic LCMV infection data at D21. **(A)** Boxplot showing the Shannon indices across cell states for each clonotype cluster. Dots color denotes cell state. **(B)** Bar plot showing TCR β chain J (left) and V gene usage (right) in each clonotype cluster**. (C)** TCR *β* CDR3 motif in each clonotype cluster.

To further characterize the clonotype clusters, repertoire analysis was conducted. Generally, divergent clones are less diverse compared to biased clones. Specifically, the Shannon indices increased monotonically from clonotype cluster 1 to clonotype cluster 4, while clones in clonotype cluster 4 have significantly higher Shannon index compared to other clonotype clusters (**Fig 5A**). In terms of clonal expansion, the clone size varies significantly both within and between clonotype clusters (**Fig H in S1 Text**). Clones in clonotype cluster1 are highly expanded, with clone size ranges from 5 to 3774 (mean 69, median 11). Clones in clonotype cluster 2 and 3 are also expanded, with clone size varying from 5 to 1004 (mean 76, median 19) and 5 to 612 (mean 27, median 11), respectively. Clones in clonotype cluster 4 are relatively small, with the mean, medium and maximum clone size being 14, 9 and 83 cells, respectively. The STATRAC clonal expansion indices also suggest that divergent clones have higher expansion capabilities compared to biased clones (STATRAC clonal expansion indices for clonotype clusters 1–4: 0.33, 0.21, 0.15, 0.06) (**Fig G in S1 Text**).

Besides, we could also observe some meaningful distinctions in the TCR *β* chain V and J gene usage across clonotype clusters (**Fig 5B**). For instance, clones in clonotype clusters 2, 3 and 4 have similar usage of TRBJ2-1, while clones in clonotype cluster 1 have little usages of this gene. On the contrast, TRBV14 was preferentially used by clones in clonotype cluster 1, while its usage in clones from other clonotype clusters are relatively low. Finally, conserved patterns, or motifs, within the TCR may provide insights into the structure, function, and antigen specificity of TCRs. We extracted the motifs of the TCR *β* chain for the top-ranked clones as an attempt to investigate the conserveness of the amino acid sequences across clones in a clonotype cluster (**Fig 5C**). Again, we could observe meaningful variations in the TCR *β* chain amino acids sequences across clonotype clusters. It will be of great interest to investigate biological implications of these variabilities in V and J gene usage and motifs across clonotype clusters.

### CD4^+^ T cell clones showed distinct phenotypic distributions along a differentiation path

**Fig 6:**
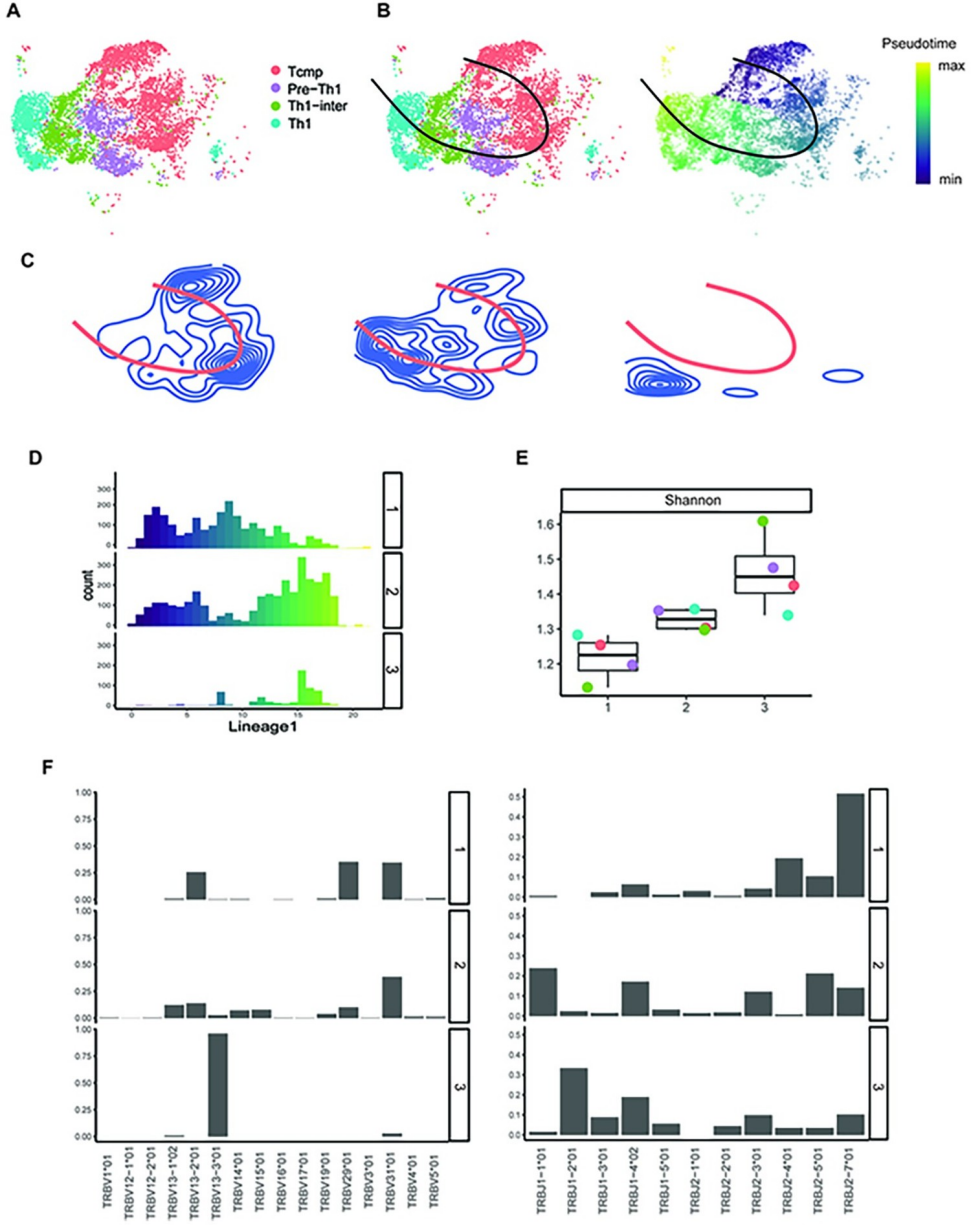
LRT analysis of CD4^+^ T cells from the mouse chronic LCMV infection data. **(A)** UMAP of the cells. Colors represent T cell subsets. **(B)** Trajectory analysis. Left: a single trajectory inferred by Slingshot; Right: pseudotime of cells. **(C)** Density contour plots for the identified clonotype clusters. Blue curves denote density contours, and orange curve represents the overall trajectory. **(D)** Histogram of pseudotime for cells in each clonotype cluster. **(E)** Boxplot showing the Shannon indices across cell states for each clonotype cluster. Dots color denotes cell state. **(F)** Bar plot showing TCR β chain J (left) and V gene usage (right) in each clonotype cluster.

As another example, we analyzed the scTCR-seq and scRNA-seq data generated from the antigen-specific CD4^+^ T cells obtained from five mice during acute lymphocytic choriomeningitis virus infection [13] (GEO accession number: GSE158896). Seurat was used to preprocess scRNA-seq data and implement UMAP dimension reduction and cell clustering. Specifically, for each sample, we filtered out doublets and dead cells (those with a percentage of mitochondrial genes > 10%), calculated cell cycle scores for the remaining cells and regressed out and normalized using the *SCTransform* approach [21]. Then, the processed datasets from five samples were integrated using the Seurat data integration workflow [20]. Finally, PCA and UMAP dimension reduction were conducted, and then cells were clustered using the Louvain algorithm [23], for which the first 20 principal components and default resolution were used. We obtained 11 cell clusters and annotated them based on the expression of well-known T cell subtype marker genes [13] (**Fig 6A**), including T central memory precursor cells (Tcmp) and the cells corresponding to different differentiation stages leading to the type 1 helper T cells (Th1), i.e., pre-Th1, Th1-intermediate (Th1-inter), and Th1. In the following analysis, we adopted this four-class expert-knowledge based annotation for cells. For scTCR-seq data, only the chains that were annotated as full-length and functional were retained. Besides, to avoid any contamination from sorting, only clones having at least two cells from a single sample were considered. After filtering, the TCR sequences from five samples were combined into a single data frame using the R package ‘scRepertoire’ [11]. The resulting scTCR-seq data was then integrated with the above-mentioned Seurat object for the remaining data analyses.

We inferred cell trajectories based on scRNA-seq data using the Slingshot algorithm [3]. Using four annotated cell types, Slingshot identified a single trajectory of Tcmp–pre-Th1 –Th1-intermediate–Th1 (**Fig 6B**), which coincides with the known trajectory of Th1 cell differentiation. Then, we identified three clonotype clusters by using DMM method (**Table B in S1 Text**), each with a different preferred residence along the trajectory (**Figs 6C** and **I in S1 Text**). We observed that clonotype clusters 1 and 3 were significantly localized in specific differentiation stages (hypothesis testing (ii) *p*-values for clonotype clusters 1–3: 0, 0.126, 0; **Fig J in S1 Text**). Clones in clonotype cluster 1 acquired all the four phenotypes, yet a higher proportion of cells are found in the earlier stage (hypothesis testing (iii) *p*-value = 0; **Fig K in S1 Text**). Clones in clonotype cluster 2 are generally concentrated on the later stage of differentiation, while for clonotype cluster 3, the proportion of later stage cells are strikingly high (hypothesis testing (iii) *p*-value = 0; **Fig K in S1 Text**). In terms of clone size, clones in clonotype clusters 1 and 2 are highly expanded, with clone size ranges from 5 to 804 (mean 48, median 11) and 5 to 762 (mean 29, median 10), respectively. Clones in clonotype cluster 3 are relatively small, where the largest clone contains 189 cells (mean 13, median 8). STARTRAC analysis also supported that clones mostly occupy the later path have lower expansion capability compared to clones that prefer the earlier path (STATRAC-expansion index of 0.36 and 0.22 for clonotype clusters 1 and 3, respectively).

Repertoire analysis further characterized the heterogeneity among the clonotype clusters. Specifically, clonotype cluster 1 that prefers early stage has lower Shannon index compared to clonotype clusters 2 and 3 that tend to later stage (**Fig 6E**). Besides, clones from these clonotype clusters showed distinct TCR beta chain V/J gene usage patterns (**Fig 6F**): in terms of V gene segment, only TRBV31-01 was shared across three clonotype clusters, yet the proportion of clones that use this gene segment in clonotype cluster 3 is extremely low. As another example, more than 75% of clones in clonotype cluster 3 use TRBV13-3*01, which was seldom utilized by clones in clonotype clusters 1 and 2. In terms of TCR beta chain J gene segment, clones from different clonotype clusters share some gene segments (e.g., TRBJ2-7*01, TRBJ2-5*01), yet the proportion still varied.

## Discussion

In this paper, we proposed LRT, a novel computational framework for investigating clonal differentiation trajectory heterogeneity by integrating scTCR-seq and scRNA-seq data. LRT addresses the limitation of previous trajectory inference methods that solely rely on scRNA-seq data and neglects the clonal relationship between cells. Specifically, LRT utilizes both the functional information from scRNA-seq data and the clonal information from scTCR-seq data. Such integrative analysis allows researchers to identify clonotype clusters with distinct phenotypic patterns along the differentiation path, which cannot be revealed solely based on scRNA-seq data. With the aforementioned strengths of the proposed LRT framework, we believe that LRT can be a powerful tool for T cell clonal differentiation heterogeneity investigation and integrative analysis of scRNA-seq and scTCR-seq data, and provide a more comprehensive view on the interplay between transcriptional regulation and T cell receptor signaling in shaping the immune response.

## Supporting information

S1 Text Supplementary information for LRT. Description of clonotype cluster level STARTRAC, additional figures and tables.
(PDF)
