# Supplementary material for: LRT: Integrative analysis of scRNA-seq and scTCR-seq data to investigate clonal differentiation heterogeneity
Source: PLoS Comput Biol. 2023 Jul 10;19(7):e1011300. doi: 10.1371/journal.pcbi.1011300 (PMC10358952; doi:10.1371/journal.pcbi.1011300)
Supplement: S1 Text — Description of clonotype cluster level STARTRAC, additional figures and tables. (PDF) [file pcbi.1011300.s001.pdf]

Text S1: Supporting Information for "LRT: Integrative Analysis of scRNA-seq and scTCR-seq Data to Investigate Clonal Differentiation Heterogeneity"

Juan Xie[1], Hyeongseon Jeon[2,3], Gang Xin[3,4], Qin Ma[2,3] and Dongjun Chung[2,3, *]

1 The Interdisciplinary Program in Biostatistics, The Ohio State University, Columbus, Ohio, USA.

2 Department of Biomedical Informatics, The Ohio State University, Columbus, Ohio, USA.

3 Pelotonia Institute for Immuno-Oncology, The James Comprehensive Cancer Center, The Ohio State University, Columbus, Ohio, USA.

4 Department of Microbial Infection and Immunity, The Ohio State University, Columbus, Ohio, USA.

* chung.911@osu.edu

A. Clonotype cluster level STARTRAC

Given a clonotype cluster with T clonotypes, the clonal expansion index, STARTRAC-expa is calculated as follows:

$$I_{expa}^{STARTRAC} = 1 - \frac{-\sum_{i=1}^{T} c_i log_2 c_i}{log_2 T}$$

where c_i is the cell frequency of clonotype i in clonotype cluster. Note that the mathematical form of STARTRAC-expa is the same as (1), yet in our case the index is defined for clonotype clusters instead of T cell clusters. The calculation of clonotype-level migration and transition indexes I_{migr}^{t} and I_{tran}^{t} are exactly the same as (1) described, yet for the cluster-level indexes, we calculate them in clonotype cluster level, instead of cell cluster level. That is, after we quantify the extent of state transition for each clonotype with STARTRAC-tran, given a clonotype cluster with T clonotypes, the clonotype level STARTRAC-tran index is calculated as the weighted average of all TCR clonotypes state transition indices contained in the clonotype cluster:

$$I_{tran}^{STARTRAC} = \sum_{t=1}^{T} p_k^t I_{tran}^t$$

where p_k^t is the ratio of the number of cells with clonotype t in clonotype cluster k to the total number of cells in clonotype cluster k, I_{tran}^t is the clonotype-level state transition index for clonotype t. Similarly, with clonotype-level tissue migration extent quantified by STARTRAC-migr, given a clonotype cluster with T clonotypes, the clonotype cluster level STARTRAC-migr index is calculated as the weighted average of all TCR clonotypes tissue migration indices contained in the clonotype cluster:

$$I_{migr}^{STARTRAC} = \sum_{t=1}^{T} p_k^t I_{migr}^t$$

B. LRT analysis of the CD8$^+$ T cells from the mouse chronic LCMV infection data at D21

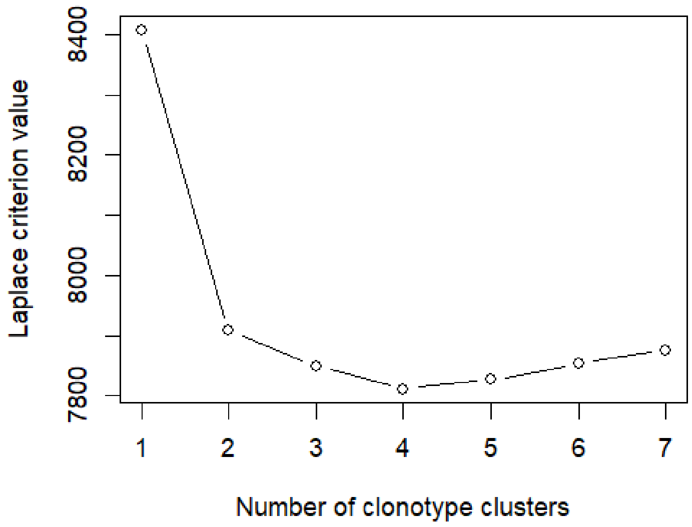

Figure A. Laplace criterion values for the DMM model as a function of the number of clonotype clusters.

Table A. The posterior mean for $\boldsymbol{p_i}$

	Clonotype cluster 1	Clonotype cluster 2	Clonotype cluster 3	Clonotype cluster 4
Naive	0.0070	0.0041	0.0044	0.0035
TEx_prog	0.1165	0.0324	0.0588	0.0062
TEx_earlyEffector	0.0463	0.0148	0.0158	0.0102
TEx_int	0.2661	0.1001	0.1859	0.0152
TEx_lung	0.1844	0.1404	0.0631	0.0081
TEx	0.3039	0.6698	0.1981	0.0068
TEx_KLR	0.0758	0.0384	0.4740	0.9500

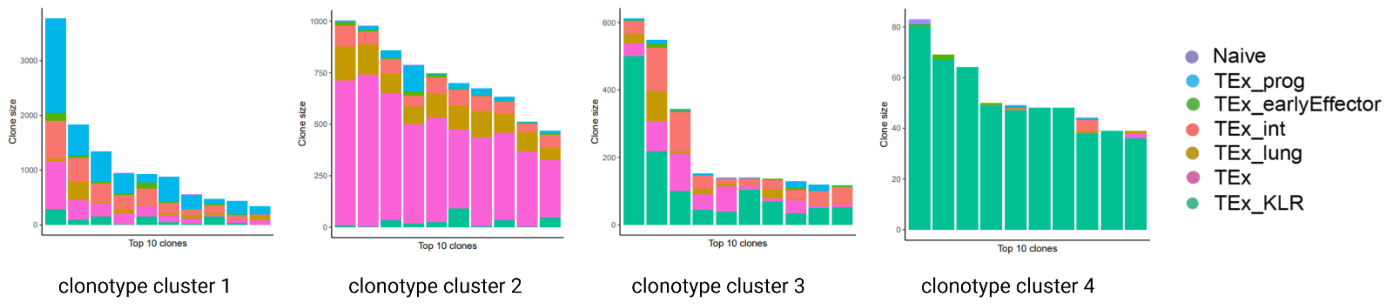

Figure B. Stacked bar plot of the phenotypic distribution of the top 10 ranked clones in each clonotype cluster.

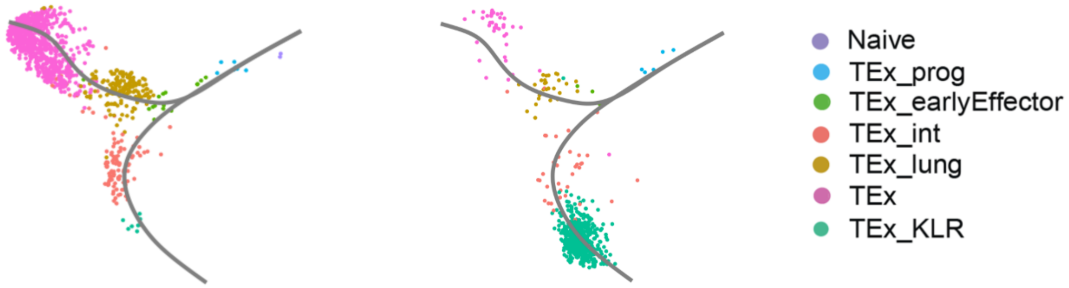

Figure C. UMAP shows the distribution of representative clones from clonotype cluster 2 (left) and 3 (right). Dots denote cells, colors denote T cell subsets, and curves denote overall trajectories.

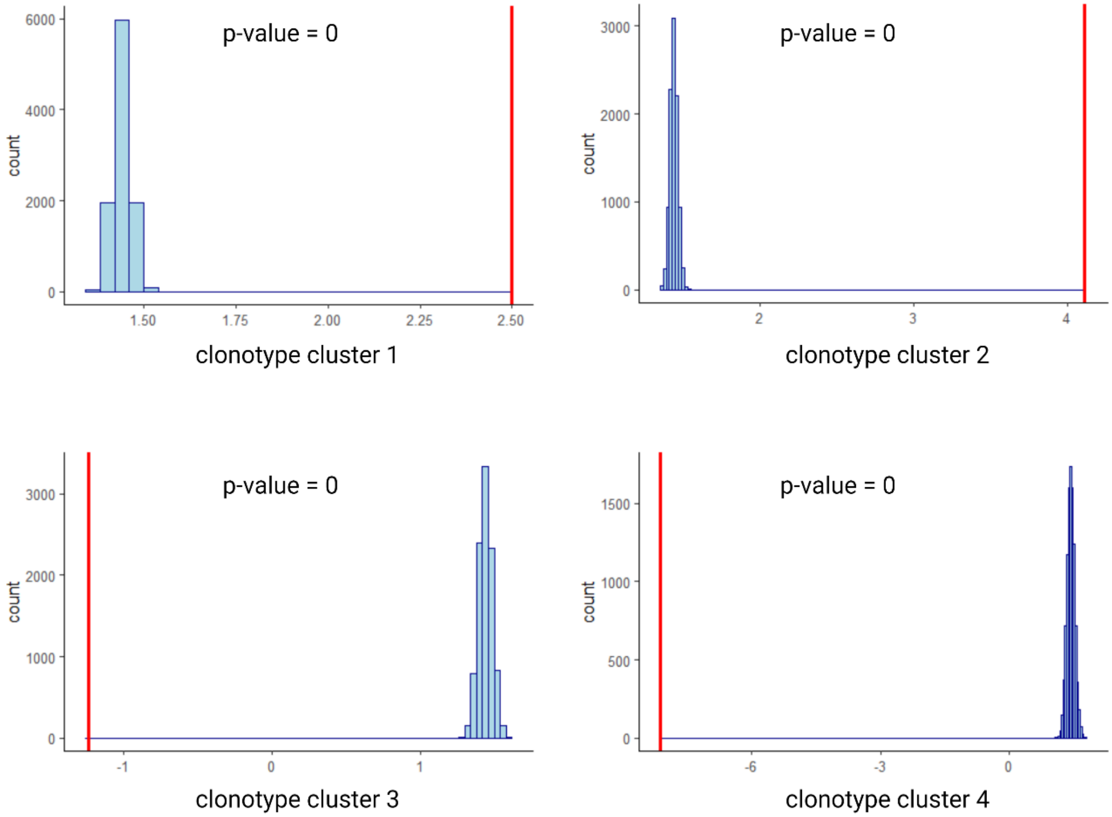

Figure D. Null distribution of hypothesis testing (i) to evaluate biased use of TEx versus TEx_KLR trajectories (# permutation = 10,000). The red lines denote observed test statistics.

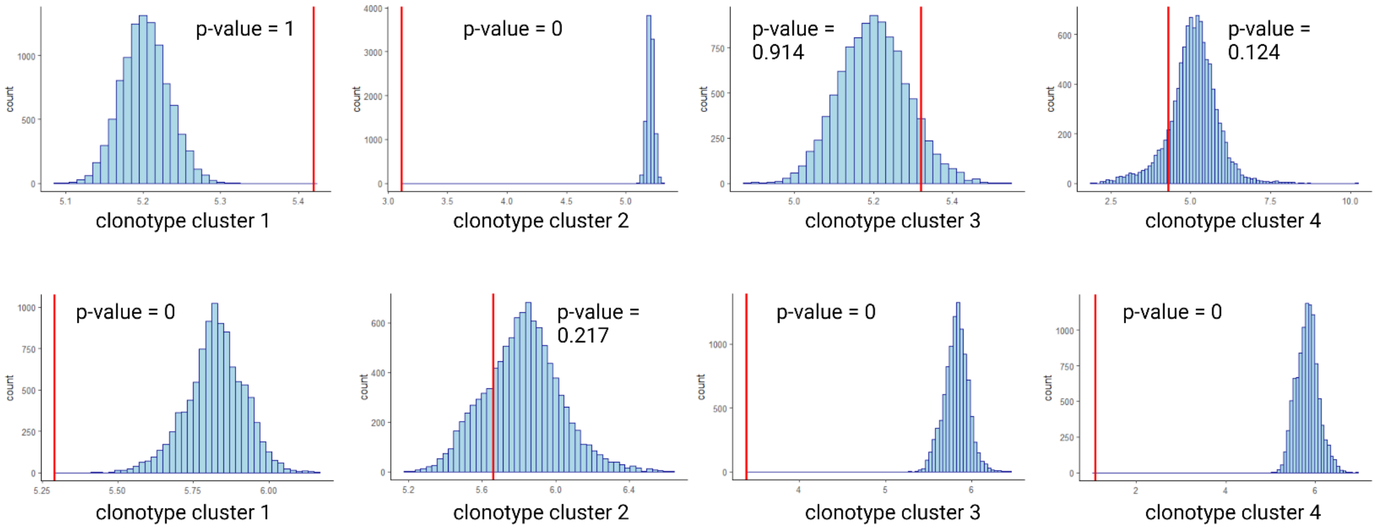

Figure E. Null distribution of hypothesis testing (ii) to evaluate the localization in a specific differentiation stage on the TEx lineage (the first row) and TEx_KLR lineage (the second row), respectively (# permutation = 10,000). The red lines denote observed test statistics.

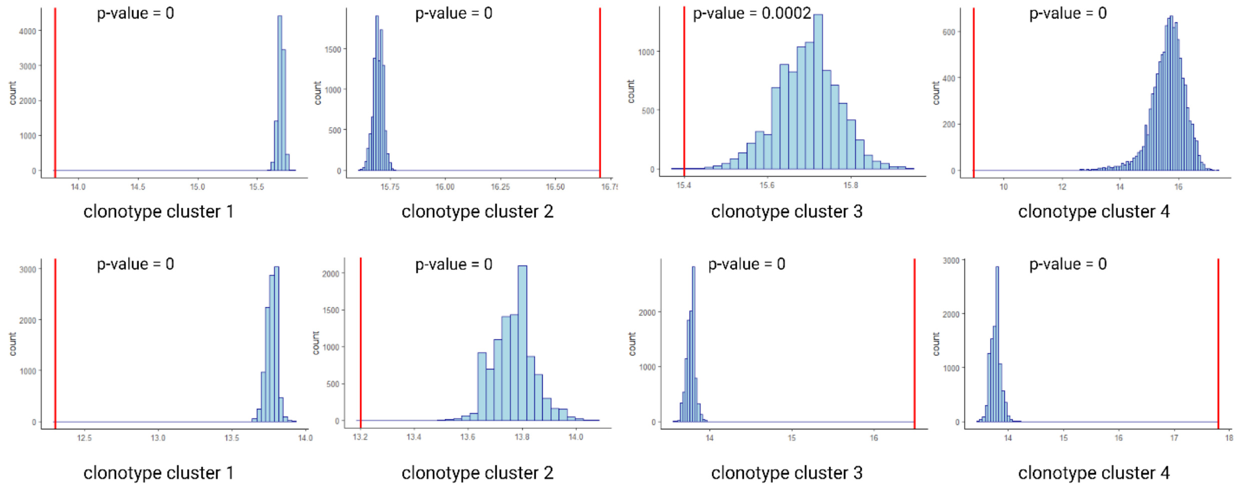

Figure F. Null distribution of hypothesis testing (iii) to evaluate the preference for earlier vs. later differentiation stages on the TEx lineage (the first row) and TEx_KLR lineage (the second row), respectively (# permutation = 10,000). The red lines denote observed test statistics.

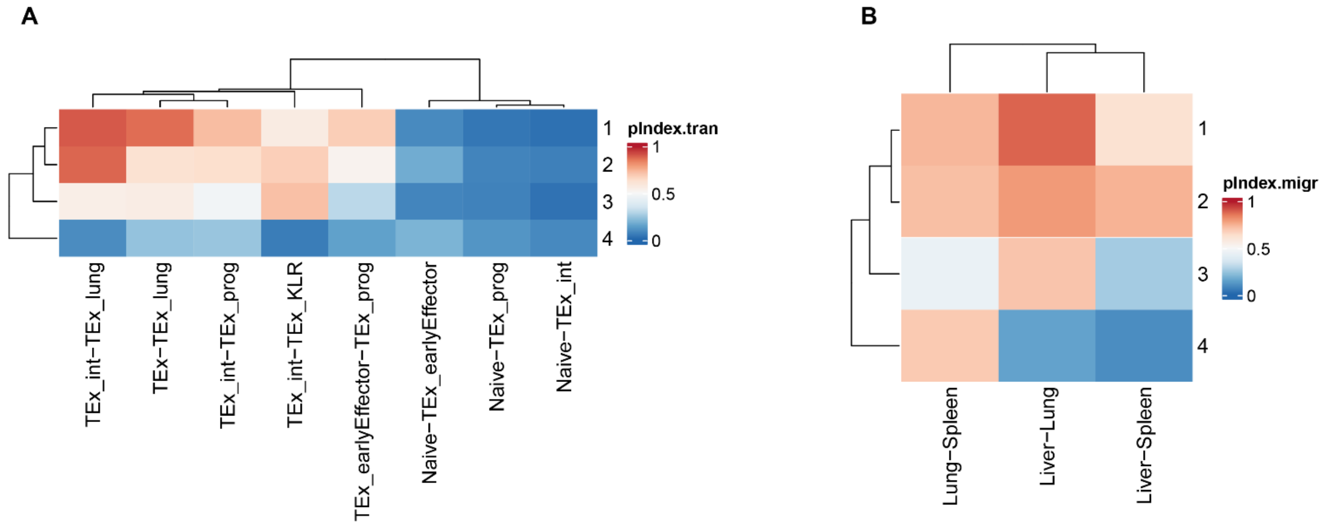

Figure G. (A) Heatmap depicting the pairwise state transition potentials for each clonotype cluster. **(B)** Heatmap depicting the pairwise tissue migration potentials for each clonotype cluster.

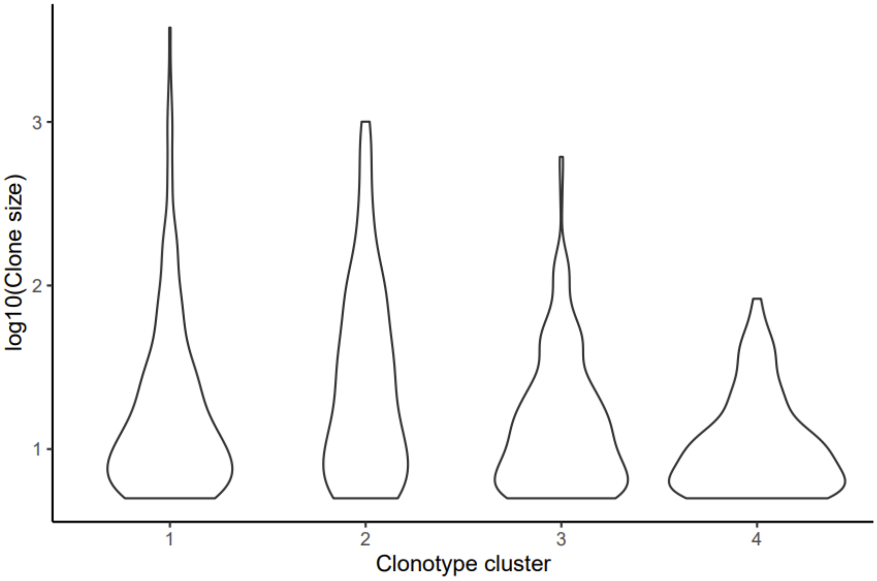

Figure H. Violin plot for the distribution of clone size for each clonotype cluster.

C. LRT analysis of $CD4^+$ T cells from the mouse chronic LCMV infection data

Table B. The posterior mean for $\boldsymbol{p_i}$

	Clonotype cluster 1	Clonotype cluster 2	Clonotype cluster 3
Tcmp	0.7404	0.3400	0.0617
Pre-Th1	0.1207	0.1859	0.2510
Th1-inter	0.0792	0.2886	0.0402
Th1	0.0597	0.1855	0.6471

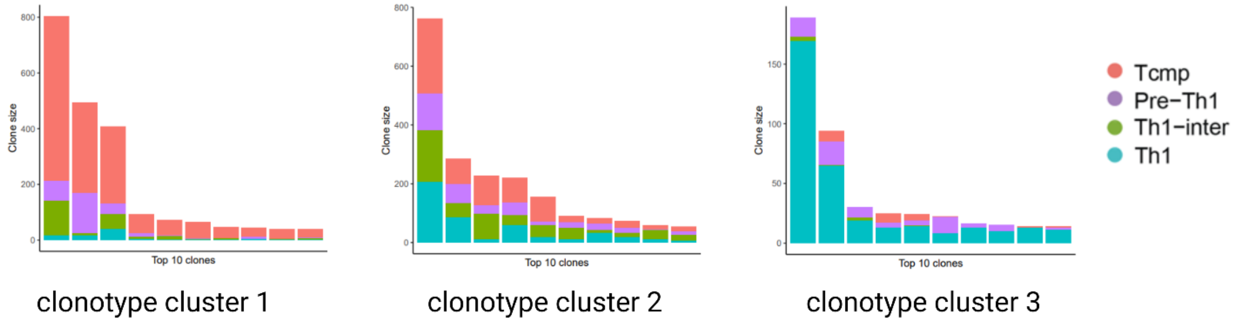

Figure I. Stacked bar plot of the phenotypic distribution of the top 10 ranked clones in each clonotype cluster.

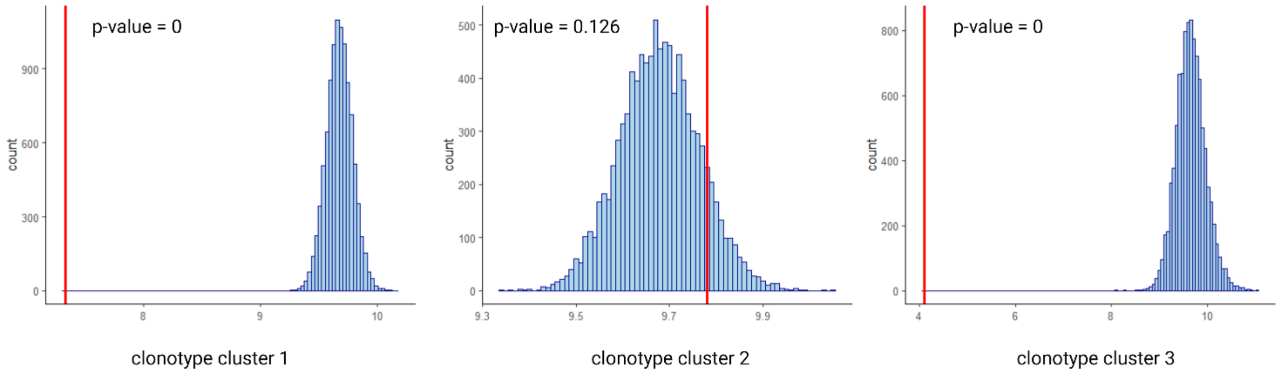

Figure J. Null distribution of hypothesis testing (ii) to evaluate the localization in a specific differentiation stage (# permutation = 10,000). The red lines denote observed test statistics.

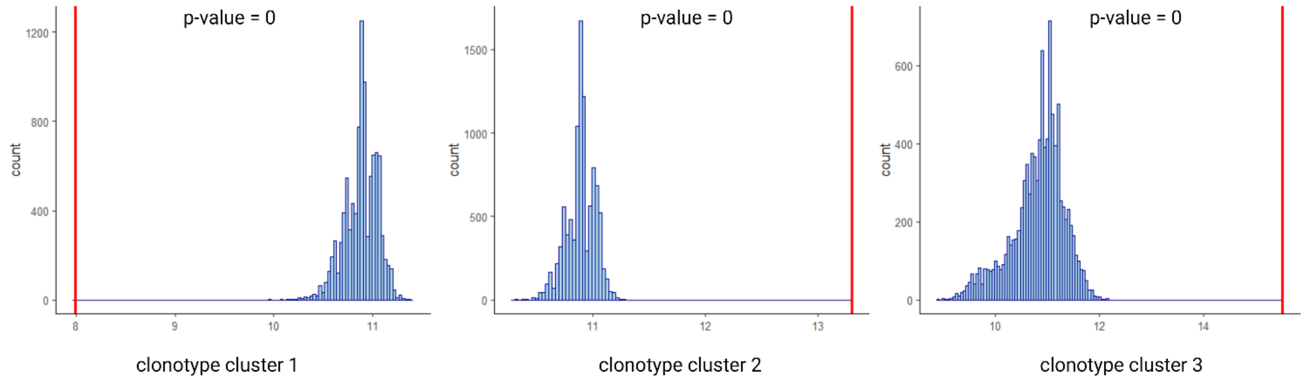

Fig K. Null distribution of hypothesis testing (iii) to evaluate the preference for earlier vs. later differentiation stages (# permutation = 10,000). The red lines denote observed test statistics.

References

1. Zhang L, Yu X, Zheng L, Zhang Y, Li Y, Fang Q, et al. Lineage tracking reveals dynamic relationships of T cells in colorectal cancer. Nature. 2018;564(7735):268-72.
